# Supplementary material for: Mg2+ Ions Regulating 3WJ-PRNA to Construct Controllable RNA Nanoparticle Drug Delivery Platforms
Source: Pharmaceutics. 2022 Jul 6;14(7):1413. doi: 10.3390/pharmaceutics14071413 (PMC9320661; doi:10.3390/pharmaceutics14071413)
Supplement: Supplementary file 1 [file pharmaceutics-14-01413-s001.zip › pharmaceutics-1738143-supplementary.pdf]

Supporting Information

Mg^{2+} ions Regulating 3WJ-pRNA to Construct Controllable RNA Nanoparticle Drug Delivery Platforms

Le Chen,[1] Jingyuan Li[1,*]

1 Zhejiang Province Key Laboratory of Quantum Technology and Device, School of Physics, Zhejiang University, Zheda Road 38, Hangzhou 310027, China; 3180103853@zju.edu.cn (L.C.)

* Correspondence: jingyuanli@zju.edu.cn

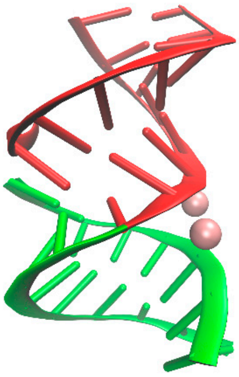

Figure S1. The schematic representation of the helix 1 (H1) and helix 3 (H3) in the 3WJ-pRNA. H1 (in red) and H3 (in green) are stacked coaxially and two pairs of bases bind Mg^{2+} ions to form two adjacent Mg clamps.

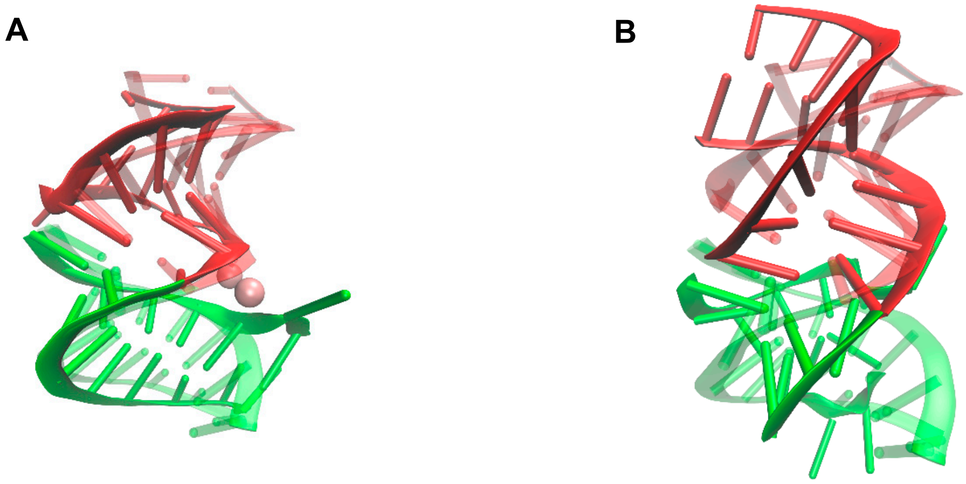

Figure S2. The schematic representation of structural changes of H1-H3 at *T* = 400 K. (A) Representative snapshots of H1 (in red) and H3 (in green) in Mg-bound 3WJ-pRNA. (B) Representative snapshots of H1 and H3 in Mg-free 3WJ-pRNA. The initial structures are shown in transparent and the final structures are shown in normal color.

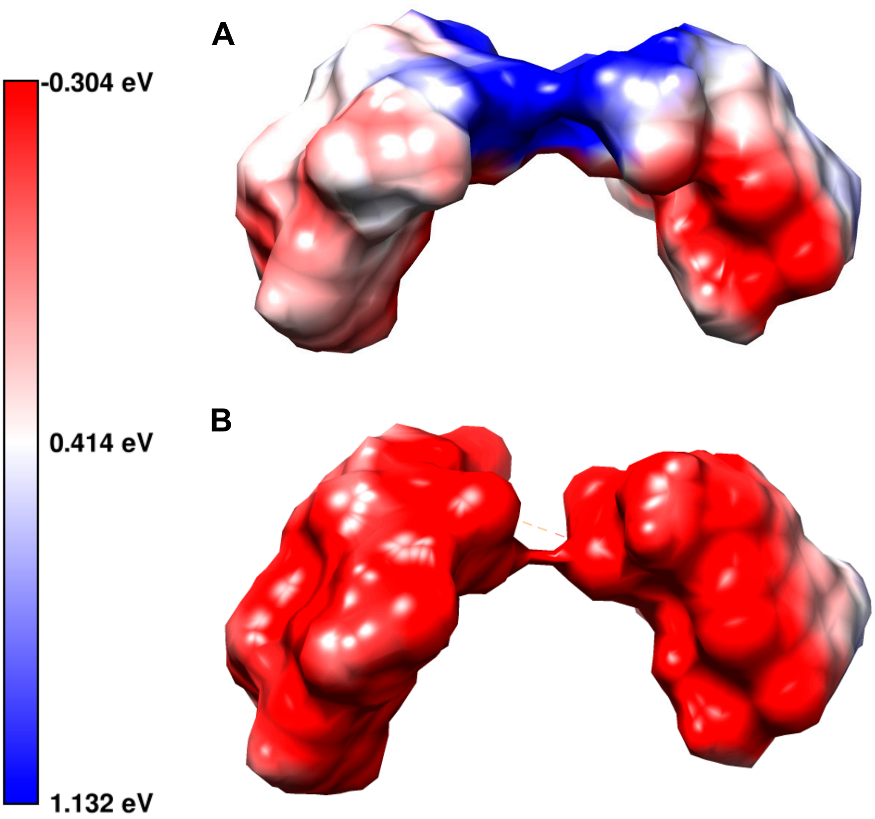

Figure S3. Mg^{2+} ions compensate the negative charges in 3WJ-pRNA backbone and reduce electrostatic repulsion. (A) Electrostatic potentials of four bases which constitute the Mg clamps, i.e., 3G, 4C of H1 and 6A, 7A of H3, in Mg-bound 3WJ-pRNA. (B) Electrostatic potentials of these bases in Mg-free 3WJ-pRNA.

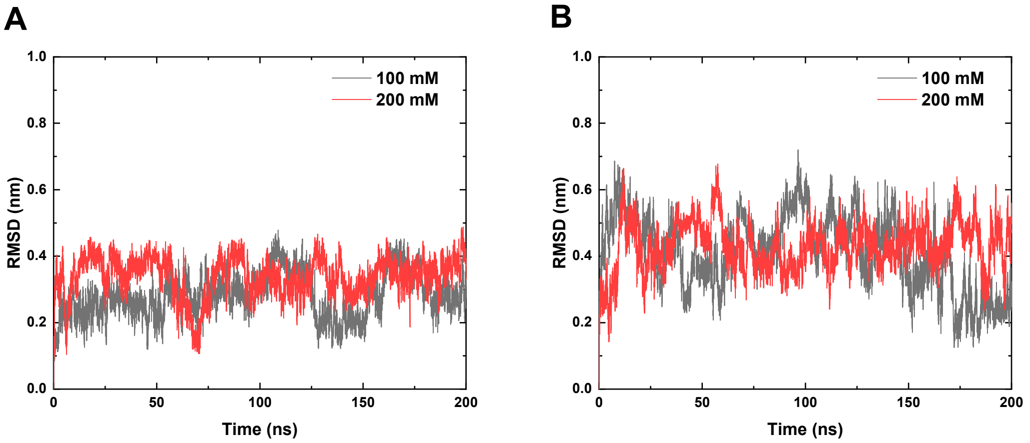

Figure S4. The RMSDs of the core region in (A) Mg-bound and (B) Mg-free 3WJ-pRNA in varying concentrations of NaCl solutions at T = 300 K.

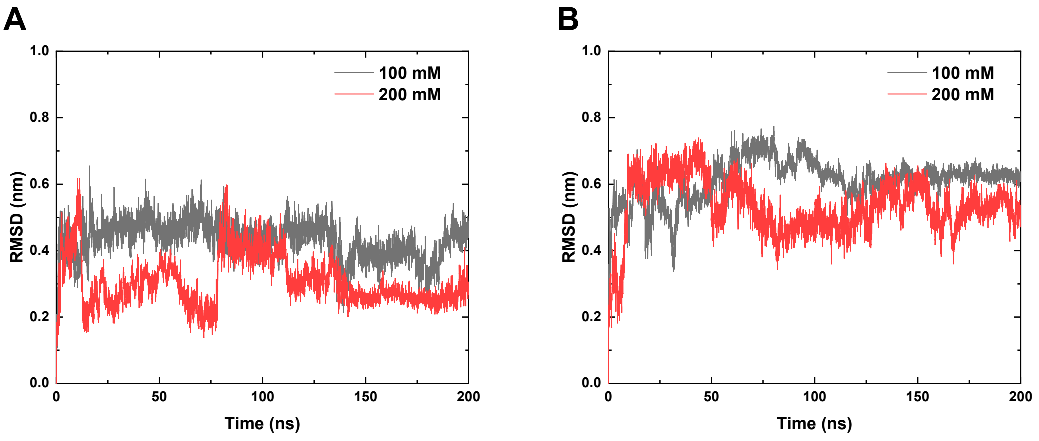

Figure S5. The RMSDs of the core region in (A) Mg-bound and (B) Mg-free 3WJ-pRNA in varying concentrations of NaCl solutions at T = 400 K.

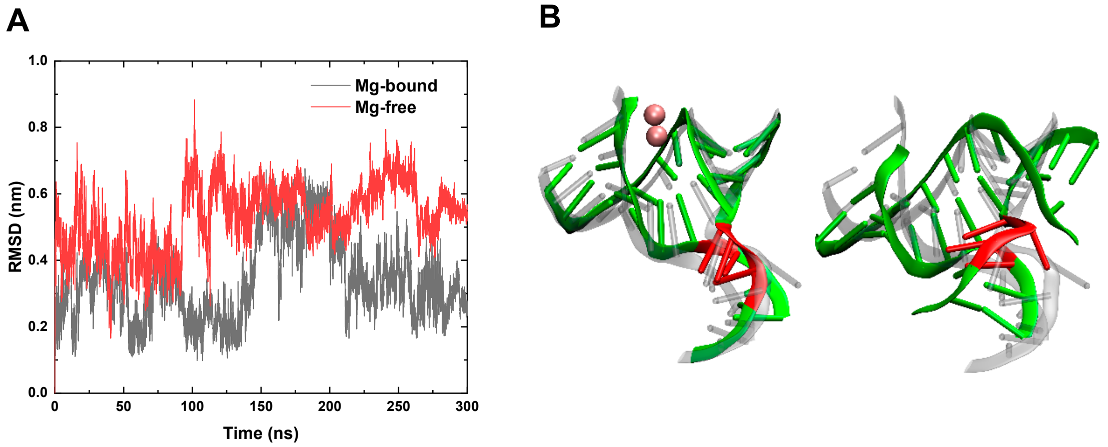

Figure S6. The structural characterizations of the core region in both Mg-bound and Mg-free systems at T = 370K. (A) The RMSDs of the core region in Mg-bound (black) and Mg-free (red) 3WJ-pRNA with respect to the initial structure. (B) Representative snapshots of the core region in the Mg-bound (left panel) and the Mg-free (right panel) 3WJ-pRNA. The initial structures are shown in transparent and the final structures are shown in normal color.

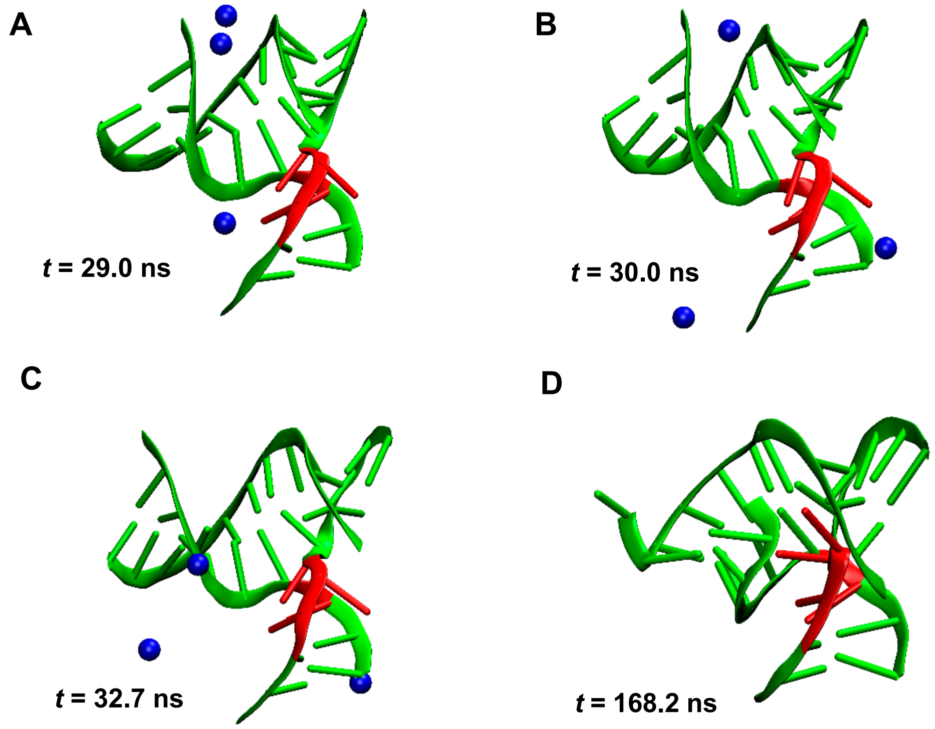

Figure S7. Na^+ ions cannot bind with 3WJ-pRNA stably. (A, B, C and D) Representative snapshots of possible binding of Na^+ ions to Mg-free 3WJ-pRNA at t = 29.0, 30.0, 32.7 and 168.2 ns under high temperature (T = 400 K).
